# Subjectivity of pre-test probability value: controversies over the use of Bayes’ Theorem in medical diagnosis

**DOI:** 10.1007/s11017-023-09614-6

**Published:** 2023-03-07

**Authors:** Tomasz Rzepiński

**Affiliations:** 1 Department of Logic and Methodology of Science, Institute of Philosophy, University of A. Mickiewicz, ul. Szamarzewskiego 89c, 60-569 Poznan, Poland; 2 Department of Biology and Environmental Protection, The Clinical Hospital of Christ Transfiguration, University of Medical Sciences, ul. Długa, Poznan, Poland

**Keywords:** Philosophical interpretations of probability, Pre-test probability value, Diagnostic process, Bayes’ Theorem

## Abstract

This article discusses the use of Bayes’ Theorem in medical diagnosis with a view to examining the epistemological problems of interpreting the concept of pre-test probability value. It is generally maintained that pre-test probability values are determined subjectively. Accordingly, this paper investigates three main philosophical interpretations of probability (the “classic” one, based on the principle of non-sufficient reason, the frequentist one, and the personalistic one). This study argues that using Bayes’ Theorem in medical diagnosis does not require accepting the radical personalistic interpretation. It will be shown that what distinguishes radical and moderate personalist interpretations is the criterion of conditional inter-subjectivity which applies only to the moderate account of personalist interpretation.

## Introduction

Rationality of the information-gathering processes is a crucial issue in all methodological analyses of medical diagnosis. The purpose of the diagnostic process is to obtain the information that is most relevant to the course of diagnostic reasoning [1, p. 996]. The information should maximize the efficiency of reasoning, thus enabling one to formulate the proper diagnosis in the shortest time possible. When choosing a test that confirms or excludes a given disease in a particular patient, the diagnostician wants to know to what extent the positive or negative test result will change the initial value of the disease probability. Some tests are better suited to confirm or rule out a particular disease than others. Rationalization of the information gathering processes requires using tools, *which allow one to evaluate the information before it is obtained*. Physicians want to know if the result of the test is potentially useful in the diagnostic process before the test is done. The diagnostician is not interested in using a test the results of which will only slightly affect the probability value of the diagnostic hypothesis. Therefore, it would be optimal to determine to what extent the result of the test planned by the diagnostician will change the probability value of the diagnostic hypothesis. In order to obtain this knowledge, Bayes’ theorem can be used. The theorem is commonly known and widely discussed by philosophers of science and epistemologists. Despite many objections, Bayesian statistics have been increasingly used in medicine, both in medical diagnosis and in evaluating clinical trials data [2–6].^1^ By using this theorem, the diagnostician can determine exactly what the value of the disease probability will be after obtaining the positive or negative test result. This means that the diagnostician can assess the usefulness of a given test (its positive or negative result) even before its conducted. In such a situation, it is possible to rationally design multi-stage diagnostic strategies.

In medical diagnosis, Bayes’ theorem allows one to determine the so-called diagnostic probability of the H hypothesis - P(H/T) - also termed as the *post-test probability* (i.e., the probability of hypothesis H in light of the test T result, where H is a diagnosis of a specified disease). However, in order to use Bayes’ theorem to calculate the post-test probability of the hypothesis one must know the *pre-test probability* value also known as the *prior probability* - P(H).

In the beginning of the 21st century, the problem of the pre-test probability estimate was recognized as a neglected area in the clinical decision-making analyses literature [7]. Currently, more and more clinicians devote their attention to this problem. The problem of determining prior probability seems to be particularly interesting from the epistemological point of view. As Mikel Aickin put it: “Because Bayesian inference is hardly ever used in disciplinary biomedical journals, it does not have as rich history to criticize. Indeed, the problems with Bayesianism come almost entirely from its philosophy” [8].

It is well-known that the pre-test probability value strongly influences the course of the diagnostic process, and its importance for the final results of diagnosis cannot be overestimated. If the value or pre-test probability for a given hypothesis is low, then the test may not have enough evidence power to change the probability value strongly enough for the hypothesis to be definitely confirmed or definitely excluded. In the case of a patient with a high pre-test probability value of the disease, the clinician can decide to use more invasive, uncomfortable, or even iatrogenic tests than in the case of a patient with a low *pre-test probability* value. Moreover, the pre-test probability value has impact on the value of the post-test probability. As Matt Bianchi has observed: “One significant barrier to routine use of probability-based test interpretation is the uncertainty inherent in pretest probability estimation” [9]. Different values of the pre-test probabilities can result in completely different treatments [10, 11]. This fact is of crucial importance for design of diagnosis.

It is generally assumed that the pre-test probability values, especially in the use of Bayes’ Theorem, are determined subjectively. The point of departure for the present article is the claim that the nature of this subjectivity should be analyzed in light of three philosophical interpretations of the concept of probability: classic interpretations based on the principle of non-sufficient reason, Richard von Mises’ frequency interpretation, and the radical personalist interpretation proposed by F.P. Ramsey and B. de Finetti. The question to be asked is how important these philosophical interpretations are for medical practice. It is obvious that for medical practitioners, philosophical questions about the interpretation of probability are clearly secondary as long as there is methodological agreement on procedures [2, p. 1061]. This seems to be the case in the use of Bayes Theorem as a support tool in medical diagnosis. Many diagnosticians are afraid of Bayesian statistics because of subjective dimension of this procedure.

The paper will argue that using Bayes’ theorem in medical diagnosis does not force one to accept the extremely radical personalist interpretation of probability. The main thesis will be that the subjectivity of the pre-test probability value in the use of Bayes’ Theorem is of an intersubjective nature. The proposed view will prove to have important consequences for medical diagnosis. The approach is based on the concept of intersubjective probability proposed by D. Gilles. In the present article, it will be argued that this interpretation is particularly appropriate for estimating the reliability of Bayesian analyses in the field of medical diagnosis. The present analysis could also enhance knowledge of philosophers and bioethicists about certain basic methodological concepts of medical diagnosis.

The considerations conducted in the article are of a prescriptive nature. Their task is to provide conditions that increase the effectiveness of the diagnostic processes carried out in medical practice. One can say that analysis describes certain idealized model of the diagnosis process, providing a pattern of solution for making diagnostic decisions.

## Various interpretations of probability

When analyzing how to assign the pre-test probability value to a diagnostic hypothesis, it is advisable to briefly discuss certain interpretations of the probability concept. Three of these have been selected for the purpose of the present considerations: a classic interpretation based on the principle of non-sufficient reason, the frequentist interpretation, and the personalistic interpretation. This distinction of the probability interpretations follows L. Savage’s taxonomy [12].

At first glance, the most appropriate approach to the pre-test probability is to use it as probability that is determined regardless of the evidence, i.e., as *a priori* probability. Justification for determining the *a priori* probability value may have its origin in two different cognitive attitudes. The first is a consequence of one’s accepting the principle of non-sufficient reason (the NSR principle). The second is founded on the personalistic interpretation of probability.

Originally the NSR principle was formulated by *inter alia* D. Bernoulli and P. Laplace. In Laplace’s account, the theory of probability consists in "…reducing all the events of the same kind to a certain number of cases equally possible […] The ratio of this number to that of all the cases possible is the measure of this probability” [13, p. 6]. In other words, if one does not know which of the events is more probable, all of them should be assigned the same probability value, the sum of which is 1. The principle has also been employed by Keynes as a basis for his logical interpretation of probability:^2^ “If there is no known reason for predicting for our subject one rather than another of several alternatives, then relatively to such knowledge the assertions of each of these alternatives have an equal probability” [14, p. 42].

The application of the NSR principle can nicely be illustrated by referring to situations such as tossing a coin, playing some dice, drawing an ace from a deck of cards, etc. In each of these cases, the probability value of individual events can be determined *a priori* without having to refer to the evidence. In these cases, the concept of probability expresses the state of one’s ignorance, but it is a state of an objectivized lack of knowledge. That one has no knowledge and one’s lack of knowledge are of the same nature because any person that understands the concept of a “coin,” “dice game,” or “deck of cards” will determine the same probability value for events on the ground of the principle of non-sufficient reason.^3^

The probability value based on the NSR principle has been subjected to severe criticism. First of all, this principle requires that considerations be limited to the situations in which one event is no more probable than others. The probability understood in this way is applicable only to trivial situations which can be qualified as “roulette-like” events. The second problem is connected with certain paradoxes that have been formulated and used to question the NSR principle.^4^

The formulation of the frequentist interpretation of probability was an attempt to overcome limitations of those approaches that were based on the NSR principle. In this account, the probability value depends on the evidence. The interpretation was proposed by Venn, von Mises, and Reichenbach. There are two main kinds of frequentism: finite and hypothetical [16].

Generally, according to frequentist interpretation, the only sense of probability is the value of number of successes in a sequence of all events. This entails abandoning the understanding of probability as a category designating an objectivized state of lack of knowledge. Agents can specify different probability values for events such as the arrival of the train on time, the loss in a gambling game, the occurrence of flu in different regions of the world, depending on the sequences of evidence that they have from the past.

The problem is that the frequentist interpretation has also several important limitations [16]. It cannot be applied to single events that have never been dealt with before. Thus, for example, in this interpretation it is not possible to determine the likelihood of encountering a foreign civilization in space. As von Mises wrote: “The probability of winning a battle, for instance has no place in our theory of probability, because we cannot think of a collective to which it belongs” [17, p. 15].^5^

The frequentist interpretation does not provide an *a priori* probability value in absolute sense, i.e., value, which is assigned independently on frequency of events on the basis of the NSR principle. On the other hand, those interpretations that accept the NSR principle allow one to determine the value of such probability, but only in relation to such trivial situations as the aforementioned roulette events. The third option is provided by the personalistic interpretation of probability.

The personalistic theory of probability was developed independently by B. de Finetti and F. P. Ramsey in the early twentieth century. According to these authors, the frequentist interpretation proposed by von Mises and the interpretations based on the NSR principle are a form of mathematical fiction, as they have no application to any decision-making situations, whether in research practice or in daily life. However, assessing the likelihood of events in a risk situation is of special importance because it stands behind every decision-making processes [12]. The concept of probability should be defined as the degree of the belief that an agent has about the event. However, the degree of the belief that an agent has about the event cannot be determined by the relative frequencies of previous events, as in the terms proposed by von Mises. Personalists assert that when making a decision in a risk situation, the agent makes in fact a certain bet in which they accept certain rates, while first analyzing the gains and losses which may be the consequence of the decision to accept the bet. “The old-established way of measuring a person’s belief is to propose a bet, and see what are the lowest odds which he will accept” [19, p. 62]. Consider a simple example to illustrate the personalistic approach.

Suppose that John and Stanley place a bet on a single roll of a dice. John bets that an even number will be cast. According to the NSR principle, both individuals determine the initial probability-value as *a priori probability* of throwing an even number as 50%, and by analogy, the same value of *a priori probability* of the opposite event. Based on the mathematical interpretations, especially the NSR principle, they would make a mistake and show their ignorance in not doing so. From the personalistic perspective, on the other hand, the probability which both individuals attribute to the events in question should be defined as the degree of the belief that can be reconstructed on the basis of the analysis made in the bets. Assume that John bet 8 euros, against Stanley’s 2 euros. John’s certainty about scoring an even number P(A) is then determined by the formula:

$${\text{P(A)}}\,{\text{ = }}\,{{\text{S}}_{\text{A}}}{\text{/}}{{\text{S}}_{\text{A}}}{\text{ + }}\,{{\text{S}}_{\text{B}}}{\text{ = }}\,{\text{8/10}}\,{\text{ = }}\,{\text{80\% }}$$

(where SA is John’s bet stake, while SB is Stanley’s bet stak﻿e.)

Thus, in terms of the personalistic approach, the probability attributable by John can be reconstructed by analyzing the rates in the bet placed by John. From the point of view of the mathematical interpretation, assigning 80% probability to an event analyzed in the situation should be treated as a bias. However, from a personalistic perspective, the value that John should attribute to the occurrence of the event, with the rules of the mathematical theory, is irrelevant. What is important is how inclined John would be to take a chance when accepting the bet. Because he is willing to gamble a lot, with relatively little profit (8 to 2), one can recognize that John is very certain about the fact that his desired outcome will occur. This means that he assigns a high probability value to the expected result.

In conclusion, according to the mathematical interpretation, John places a bet because the pre-test probability is 50%. In turn, on the basis of the personalistic interpretation, the pre-test probability is 80% because John decided to accept the bet on certain conditions (i.e., at the specified stake values). It can, therefore, be said that in the frequency interpretation, the pre-test probability is the starting point for making the decision to participate in the bet, while in the personalistic interpretation the pre-test value of probability is constituted on the basis of an estimate of the winning chances. The problem is, however, that it is likely to be understood in an extremely subjective way. On the epistemological level, the personalistic interpretation remains in opposition both to the NSR principle and the frequency interpretation.

It is worth noting that the personalist interpretation permits one to assign *a priori* probability values based on a risk estimate even to a single event or to one that has never occurred before. The issue of the single event probability has been widely discussed in philosophical literature in the context of the reference class problem.

## The problem of pre-test probability estimates in medical diagnosis

Now, consider why the determination of pre-test probability is important for the course of diagnostic process. The first argument is that prior probability value affects the course of diagnostic inference in the sense that if the value for a given hypothesis is small, then a test with average evidential power will not change this value significantly enough for the hypothesis to be definitely confirmed or definitely excluded.

However, in medical practice, tests with high evidential power are also used; tests perceived as conclusive constitute the gold standard. They often play the role of reference tests which constitute the criterion for the evaluation of other diagnostic tests. It would seem that the value of the pre-test probability of disease occurrence in case of such a tests is of little importance. The result of the high-evidence test is considered to be ultimately conclusive. Nevertheless, the value of pre-test probability may also be of paramount importance for the course of the diagnostic process in this situation. For example, it is well known that coronary angiography is a test of a high evidential power in the diagnosis of certain types of coronary artery disease (CAD). In the case of a patient older than 60 with obesity and characteristic chest pain, coronary angiography will probably be ordered because of the high pre-test probability of CAD. However, in the case of a 22-year old man with the same symptoms, the diagnostician may refrain from recommending such a test, because of the low pre-test probability of CAD and because invasive character of the test. It is clear that the main issue is not the possibility of performing a conclusive test. The problem is whether diagnostician identifies indication for this test on the bases on their knowledge about priors, which values are determined due to reference classes (like age and obesity in the above example).

The problem of determining the pre-test probability values in medical diagnosis may appear in the context of three issues: (i) reference class problem(s), (ii) mentioned above doctors’ different clinical experiences, and (iii) differences in perception.

Consider more precisely the first issue. Suppose that a diagnostician knows, on the basis of some epidemiological data, that the probability of a coronary artery disease (CAD) for the patient with specific characteristics: age, weight, low-density lipoprotein (LDL) high level, is equal to *p*. In this case, the epidemiological research constitutes a reference class for the patients with the given characteristics. The problem is that in the medical diagnostic process, doctors have to consider patients who share these basic characteristics but differ in additional features not included in this epidemiological study. For example, they live in different countries, they have different food habits, and so on. In such situations the pre-test probability of the CAD for a given patient is a single event probability, which is altered depending on the additional features of the patients. The question is whether in such cases one should accept the personalist interpretation of probability or whether one should recognize that probability value cannot be assigned for single events. The latter solution is accepted, among others, by von Mises. A compromise solution has been proposed by D. Gillies, who argues that single events probabilities are of subjective nature, but they are based on objective probabilities. According to Gilles, first, an event must be assigned for the reference class for which there are good statistical data. This allows one to calculate the objective probability. In the next step any further information which is relevant for the occurrence of the event should be taken into account. Consequently, the information gathered makes it possible to adjust the objective probability value down or up [20].

There are few important questions in the context of Gillies’ proposal. The first one is what kind of justification should be given for the probability adjustment - one’s individual clinical experiences, the colleges’ opinions, the consistency with the basic scientific knowledge, or financial analysis based on the *number needed to treat* parameter? The second question concerns the problem of the probability adjustment limitation. It is easy to imagine that individual clinical experiences allow one to adjust the probability value in completely different manners so that the probabilities of disease disorder assigned by different diagnosticians can vary in significant way [10].

The second problem of pre-test probability estimates is the doctors’ different clinical experiences. When formulating their diagnostic hypotheses, doctors rely on their previous experience which involves similar occurrences of symptoms in patients that they have examined. C. Scandellari writes, “As clinical experience evolves, our case list is continually updated in memory and calibrated directly to our local practices (…) From our memories, commonly diagnosed causes […] may be readily recalled, while less common disorders may be retrieved more slowly…” [21, p. 387].^6^

The third aspect of the problem of pre-test probability estimation is connected with the diagnostician’s skills [23, p. 891]. The pre-test probability values are estimated by a physician that is already at the first stage of their diagnosis, i.e., the interview and physical examination. At the stage of physical examination, it is of particular importance for assessing the probability value to specify the diagnostician’s predispositions in terms of listening to the murmurs and noises in the lungs and heart, palpably sensing the organ disorders, differentiating tapping sounds or diversifying of skin rash, and so on. Some doctors show greater skills in this respect, while others are less skilled [24]. These skills are also subject to development and they contribute to the physician’s “expertise knowledge” [23, 24].

Different perception skills can cause doctors to assign different pre-test probability values. A doctor that cannot distinguish the physiological murmur of tricuspid in children from general pathology does not have a sufficient basis for formulating the hypothesis that identifies the patient’s heart defect. Research shows that both a novice and an experienced physician often attribute different pre-test probability values even in relatively simple clinical situations which involve determining the probability on the basis of basic information obtained in the interview or during the physical examination [7, 10, 25].

In the above case, the subjective probability value is not, however, a consequence of rejecting the frequentist interpretation of probabilities, but a consequence of not having the necessary data. If a hearing impaired or an untrained diagnostician were able to listen to the non-physiological tricuspid murmur, they would probably assign a high-value probability to the hypothesis that the patient’s heart defect has been caused by tricuspid [26].

## Grades of subjectivity in probability estimates

The present paper suggests that subjectivity of the probability value has different dimensions and must be clearly distinguished. Consider a simple example in order to illustrate this. Suppose that John and Mary go to work on the morning bus number 38. John goes to work from point A to B, while Mary from point B to A. John is never late to work, because the driver of the morning bus is deeply committed to be on time. Mary has a completely different experience of commuting to work from point B to A; this bus is never on time. These two persons will assign different probability values to the bus 38 punctual arrival event. In this situation, however, is it really legitimate to speak about the personalistic interpretation of the probabilities in terms of Ramsey’s and de Finetti’s approach? Certainly not. The subjective nature of the probability value is simply a consequence of the agents’ having different experiences. These, however, are, (at least potentially) intersubjectively verifiable by another agent. There is nothing that prevents John and Mary from recognizing the probability values they have assigned if they are specified for the opposing bus directions.

Suppose that after passing point B bus 38 goes to Airport C. One time John decided to spend vacation in the Caribbean and booked a flight from airport C. The departure from the airport takes place in an hour, which allows John to get to the airport by bus number 38. The likelihood of a punctual arrival is defined as the relative frequency of the events based on John’s experience. Thus, there is no reason to believe that the bus will be late and will not arrive at the airport on time. Despite the very high value of the frequency probability, John, due to the high cost of the airline ticket, decides not to take the risk and orders a taxi. He believes that this is a more reliable transport that will guarantee that he is not late for the flight.^7^

The above example represents a situation in which a subjectively attributed probability value is not only the result of different experiences, but, as radical personalists say, it is also designated as a function of beliefs that determine the decisions in a risk situation. John estimates the benefits of getting a bus (a low fare ticket) and as he compares this benefit to the potential loss (the cost of flight and the loss of a paid-for stay in the Caribbean), he recognizes that the choice of a bus as a means of transportation is subject to too much risk. This example gives a clear indication of the reason why the personalists have found the mathematical interpretations of probabilities to be useless fictions, not applicable to analyses of the actual decision-making processes. The basis for the decision-making process is always the estimate of the potential costs and losses that result from the decision made in a risk situation.

The above example shows the need to distinguish between two dimensions of subjectivity in the process of assigning the probability value. The first one, relatively uncontroversial, is the consequence of the agents’ having different experiences. The second one is connected with the radical personalistic interpretation. I will now consider the first one more closely.

## The concept of intersubjective probability

The problem of subjectivism in pre-test probability of diagnostic hypotheses assignment is one of the most crucial issues in discussions of the medical decision making process. However, it would be a mistake to treat all clinical situations in the same way, as this would entail that considering that the radical personalist interpretation of probability is the only appropriate approach. When analyzing the problem of the pre-test probability value, it is advisable to clearly define a *moderate personalist account* which is connected with the *intersubjective probability* concept.

The idea of intersubjective probability was originally proposed and developed by D. Gillies [15, 28]. The concept is characterized in the following manner:

If a group (of people, doctors, scientists and so on – [T.R.]) does in fact agree on a common betting quotient, we shall call that betting quotient the *intersubjective* or *consensus* probability of the social group. This type of probability can then be contrasted with the *subjective* or *personal* probability of a particular individual. [28, p. 517].

There are two conditions that must be fulfilled in case of group of people who share probability value in the intersubjective way.

The first one is the condition of common interest. According to it, the members of the group must be united by a common purpose, which can be of financial, medical or of any other nature. The second condition is that of the information flow, according to which, information that is relevant for the common purpose has to be transmitted to all members of the group [28, p. 518].

This paper argues that it useful to adopt a form of Gillies’ intersubjective probability for the purpose of analyzing various cases of pre-test probability of diagnostic hypotheses estimation. The thesis is that two types of subjectivity in the field of medical diagnosis should be distinguished. In the first one, the subjective dimension of probability is reconstructed in the category of individual assessment. This individual assessment is obviously influenced by characterological predispositions of the doctors, their propensity to take risk. In this case, the radically personalistic interpretation of probability is assumed. A characteristic feature of this interpretation is that the agent’s belief(s) will not necessarily be embraced by other members of the social group, i.e., other clinicians, doctors and so on.

I will illustrate the above claim with the aforementioned example of John and Mary. Suppose that John decides to go on his first life flight to Caribbean despite the fact that he is not very rich. He is very afraid of losing money and he decides to be at the airport 7 hours before the departure of the plane. By contrast, Mary is very rich and has travelled to numerous parts of the world. Being late for the flight would not be such a painful disappointment for her as it would be for John. John’s fear of missing the flight would be incomprehensible for Mary. Sharing common beliefs is not possible, because, despite the information flow, the obtained data are not accepted by both subjects. The conclusion is that a key point of the *intersubjective probability* concept proposed by D. Gillies is one’s *openness for data acceptance*. Person A questions the value of *p* because they are afraid that accepting this value on the basis of knowledge about the event’s frequency is a cognitive error that may have adverse practical consequences in terms of decisions and actions taken. It was just the case of John, who was not open to accepting the probability of the bus punctuality and decided to violate the intersubjective probability condition.

Now, I will apply the above findings to a situation in which the pre-test probability value is determined in the Bayesian Theorem used for assessing the informativeness of medical diagnostic tests.

## The diagnostic test’s parameters in the use of Bayes’ Theorem in medical diagnosis

Formal analyses of the diagnostic process create models that provide a better understanding of what kind of actions the diagnostician should undertake to increase the effectiveness of the diagnostic inference. They do not describe any realistic diagnostic procedures. Rather, they provide some patterns of good practice for the selection of diagnostic tests with given evidential power. Therefore, the created models are of prescriptive nature. However, do these models have a real impact on the diagnosis process carried out in medical practice? They definitely should. The diagnostician must know what parameters characterize tests with a high ability to confirm or reject a given hypothesis, because these features are independent of each other. The high ability of a given test to confirm the disease does not prove that the test is equally well suited to the exclude the disease and vice versa. Thanks to the knowledge about the test parameters, the doctor can select the diagnostic tools consistent with the goals realized in the given diagnostic procedure. The educational aspect of increasing the awareness of the clinicians about statistical concepts and the models created with them is implemented in the field of EBM training for doctors.

However, formal analyses also provide models for the development of the general guidelines for the clinical decisive rules in differential diagnosis. A good example is the guidelines for the differential diagnosis of CAD formulated by European Society of Cardiology [29]. In this case, the clinical guideline model was developed on the basis of the findings about prior probabilities of the different types of cardiovascular disease, based on the patients characteristics and symptoms. The clinical guidelines precisely define the way of dealing with the specific groups of patients, determining what kind of tests should be applied to these patients at the subsequent phases of diagnostics. These guidelines were developed on the basis of the formal findings describing both the process of assigning prior probabilities for specific patient subpopulations and the knowledge about parameters of the diagnostic tests, which are used in the differential diagnosis of CAD. I will now look more closely at the general formalism underlying the development of these kinds of guidelines.

Suppose that, on the basis of an interview and physical examination of a patient, the diagnostician has formulated some preliminary diagnostic hypotheses H_1_, … H_n_. Every of these hypotheses predetermines different diseases for the given patient. Further, assume that the probability values can be assigned for every of the hypotheses, (i.e., P(H_1_) = p_1_, … P(H_n_) = p_n_) on the basis of epidemiological studies. These are the pre-test probabilities. In the next stage of the diagnostic process, the physician will try to obtain information from additional diagnostic tests (e.g., laboratory, biophysical, and so on) that will change the pre-test probabilities of the hypotheses. As a result, some hypotheses will be excluded from the set in question, while others will become viewed as more likely. For the purpose of analyzing the diagnostic decision process, it has been proposed in the literature that one should use the so-called *threshold approach* [30]. According to this proposal, two thresholds for the decision process of diagnosis can be approximately assigned. The first one is the *treatment threshold* which determines the minimum probability value of a hypothesis the diagnostician recognizes as sufficient to confirm their disease hypothesis and apply the appropriate therapy. The second one is the *test threshold* which establishes the minimum probability value below which the diagnostic hypothesis is recognized as refuted. These two thresholds are separated from each other by a testing zone, the so-called *uncertainty area*. Tests are performed for hypotheses in this area. Scheme 1

**Figure:**
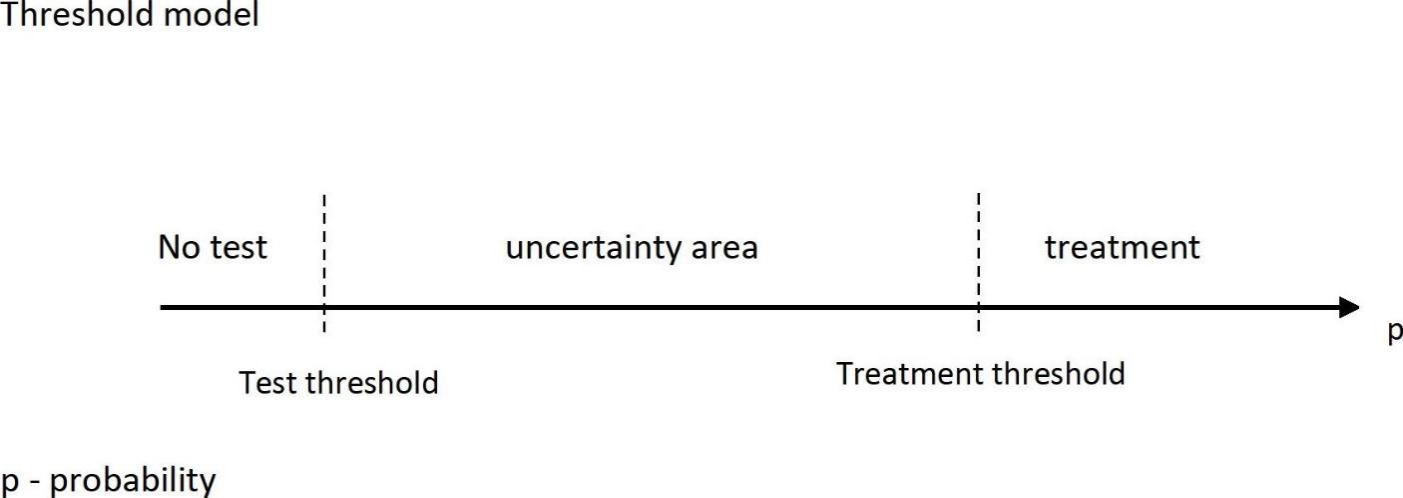
**Scheme 1**

It has to be noted that the thresholds have no absolute values since their values depend on many different factors of medical, social and economic nature. For example, the treatment threshold is influenced, among others, by the iatrogenic nature of the adverse effects. The higher the degree of diagnostician’s belief in the side-effects caused by a treatment, the higher the probability value of the treatment threshold [31, p. 2]. Similarly, the value of the test threshold depends on the predictions established for patients with regard to a disease. The more damaging the effects of a disease are, the lower the test threshold is. Thus, in general, the “testing zone is a function of the test properties risk” [32, p. 406]. Whether the diagnostic procedure will primarily aim at the confirmation of a particular diagnostic hypothesis depends on the clinical situation identified by the type of disease, its risk, the information obtained in the first stages of diagnosis (interview and physical examination), and other elements that constitute the knowledge of the diagnostician [33].

Suppose that, initially, the diagnostician considers making a diagnostic test T_*α*_, that can change the probability value of the diagnostic hypothesis H^+^. The test confirming this hypothesis (positive) will be marked as T_*α*_^+^, while the test that refutes this hypothesis (negative) as T_*α*_^−^. Before the diagnostician performs the test, they try to determine the extent to which the T_*α*_^+^ or T_*α*_^−^ result will change the pre-test probability value of hypothesis H^+^. This means that they want to calculate the possible post-test probabilities of hypothesis H^+^ in the face of both potential results of the test.^8^ Narrowing the consideration to the positive result of the test, the question becomes: how much the value P(H^+^) will change after the result T_*α*_^+^? In other words, what is the P(H^+^/T_*α*_^+^) value? The answer can be obtained by using Bayes’ Theorem in the following form:

$$P({H^ + }/{T_\alpha }^ + ) = \frac{{P({H^ + }) \times P({T_\alpha }^ + /{H^ + })}}{{P({H^ + }) \times P({T_\alpha }^ + /{H^ + }) + P({H^ - }) \times P({T_\alpha }^ + /{H^ - })}}$$

where P(T_*α*_^+^/H^+^) is the so-called diagnostic sensitivity of the test (D.Sen.), whereas P(T_*α*_^+^/H^−^) = 1 – diagnostic specificity (D.Spec.) of test T_*α*_.

Here, I introduce both these concepts (diagnostic sensitivity and diagnostic specificity) but without discussing the specific epistemological issues that are entailed by them.

Both parameters, i.e., the diagnostic sensitivity and the diagnostic specificity, are discussed in the studies that aim to determine the accuracy of the index test (or evidence value of the test) compared to the so-called reference test.^9^ The reference test is a criterion that allows one to recognize the truth or falsehood of the index test result. Consequently, one can obtain four potential results of the index text regarding the reference test: true positive results (TP), true negative results (TN), false positive results (FP) and false negative results (FN). 10 These findings are clearly presented in the scheme below. Scheme 2

**Figure:**
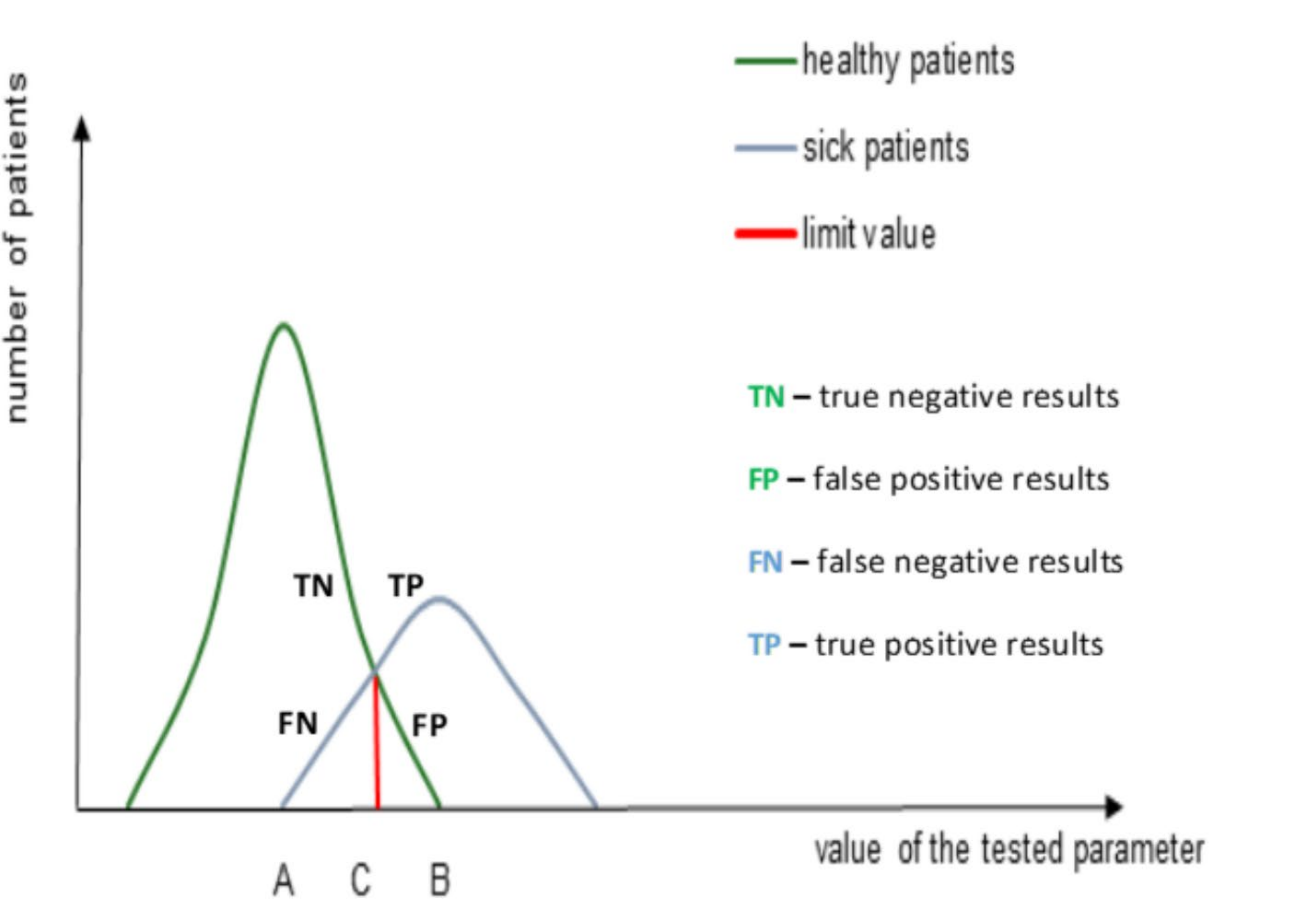
**Scheme 2**

In brief, TP are the results of a test that correctly identifies a disease in a person that is ill. Analogically, FP are the results of a test that incorrectly attributes a disease to a person that is healthy.^11^ It is similar in the remaining cases of the TN and FN results. These findings allow one to characterize the concepts of diagnostic sensitivity and diagnostic specificity in the following manner.^12^

Generally, the diagnostic sensitivity of the index test means the ability of the test to recognize the disease in the set of sick people. Thus, it is the probability of the positive test result in disease patients P(T^+^ / H^+^).

$$D.Sens = P({T^ + }/{H^ + }) = \frac{{TP}}{{TP + FN}}$$

Accordingly, the diagnostic specificity of the index test is the test’s ability to rule out the possibility of a disease in a set of healthy people. Thus, it is the probability of a negative test result in healthy patients P(T^−^ / H^−^).

$$D.Spec = P(T^-/H^-) = \frac{TN}{TP + FN}, Because{\rm{ }}P\left( {{T^ - }/{H^ - }} \right){\rm{ }} + {\rm{ }}P\left( {{T^ + }/{H^ - }} \right){\rm{ }} = {\rm{ }}1,{\rm{ }}then{\rm{ }}P\left( {{T^ + }/{H^ - }} \right){\rm{ }} = {\rm{ }}1{\rm{ }} - {\rm{ }}P\left( {{T^ - }/{H^ - }} \right)$$

Taking into account the above, Bayes’ Theorem for the case in question can be represented in the following manner:

$$P({H^ + }/{T_\alpha }^ + ) = \frac{{P({H^ + }) \times D.Sens}}{{P({H^ + }) \times D.Sens + P({H^ - }) \times (1 - D.Spec)}}$$

where value P(H^−^) = 1 – P(H^+^)^13^.

When one wants to use the Bayesian Theorem in evaluating the data that the diagnostician aims to obtain, it is necessary to know the diagnostic sensitivity and the diagnostic specificity of the test and the pre-test probability value of the diagnostic hypothesis. From the perspective of a philosophical analysis, certain very interesting epistemological problems arise both in relation to the process of determining the diagnostic sensitivity and the diagnostic specificity as well as in relation to the process of determining the pre-test probabilities of hypotheses [38]. The issues considered in this paper will be limited to the latter group of problems.

## Bayes’ Theorem in designing diagnostic strategies

In the subject literature, it is usually emphasized that using Bayes’ Theorem involves interpreting the pre-test probability in a fairly subjective way. From the perspective of epistemological considerations of medical practices, there arises the question regarding the degree of subjectivity in using Bayes’ Theorem in medical diagnosis.

When using Bayes’ Theorem, the diagnostician can consider different diagnostic strategies by selecting the one that provides accurate diagnosis in the shortest time possible. Suppose that on the basis of an interview and physical examination, the doctor formulates a single H^+^ diagnostic hypothesis about the occurrence of disease D in the patient. For the sake of this example, further assume that for the first step of the diagnostic process the value of the pre-test probability P(H^+^) is determined on the basis of epidemiological studies that specify the frequency of a given disease in a population with the characteristics and symptoms similar to the symptoms and characteristics of the patient considered by the diagnostician. One of the most commonly accepted methods is to calculate the so-called probability of the target disorder or prevalence parameter [33, p. 442]. It represents the ratio of the number of all ill patients who have been tested in the index test to the number of all healthy and sick patients tested. See below.

$$P\left({H}^{+}\right)=\frac{TP+FN}{TP+FN+TN+FP}$$

Suppose that on the basis of an epidemiological study, it is ascertained that P(H^+^) = 15%.^14^ Furthermore, assume that the aim of the strategy that the diagnostician employs is to confirm the hypothesis in such a way that the treatment threshold, which is 90%, has been exceeded. This means that the doctor will assume that the diagnostic process has ended with diagnosing the disease and the appropriate treatment should be implemented when the value of the diagnostic hypothesis is 90% or more. To confirm the hypothesis of H^+^, the physician considers the performance of T_*α*_ test, whose diagnostic sensitivity is 67% and whose diagnostic specificity is 78%. By using Bayes’ Theorem, the diagnostician can easily determine that the positive result of the test will yield the increased probability of the disease in a specific patient from 15 to 35%, as presented below.

$$P({H^ + }/{T_\alpha }^ + ) = \frac{{P({H^ + }) \times D.Sens}}{{P({H^ + }) \times D.Sens + P({H^ - }) \times (1 - D.Spec)}}$$

$$P\left({H}^{+}/{T}_{\alpha }^{+}\right)=\frac{\text{0,15}\times \text{0,67}}{\left(\text{0,15}\times \text{0,67}\right)+\text{0,85}\times (1-\text{0,78})}=35\%$$

However, in the design of diagnostic strategy, the physician has to take into account the possibility that result of T_*α*_ test will be negative. Thus, they must transform Bayes Theorem to the appropriate form so as to calculate P(H^+^/T_*α*_^−^). See below.

$$P\left({H}^{+}/{T}_{\alpha }^{-}\right)=\frac{P\left({H}^{+}\right)\times P({T}^{-}/{H}^{+})}{P\left({H}^{+}\right)\times P({T}^{-}/{H}^{+})+P\left({H}^{-}\right)\times P({T}^{-}/{H}^{-})}$$

It is easy to notice that if P(T^−^/H^+^) + P(T^+^/H^+^) = 1, then P(T^−^/H^+^) = 1 – sensitivity. See below.

$$P\left({H}^{+}/{T}_{\alpha }^{-}\right)=\frac{P\left({H}^{+}\right)\times (1-sensitivity)}{P\left({H}^{+}\right)\times (1-sensitivity)+P\left({H}^{-}\right)\times specificity}$$

Finally, one can easily determine that the negative result of the T_*α*_ test will decrease the probability of the disease in a specific patient from 15% to 6,9%, as presented below.

$$P\left({H}^{+}/{T}_{\alpha }^{+}\right)=\frac{\text{0,15}\times (1-\text{0,67})}{\text{0,15} \times (1-\text{0,67})+\text{0,85}\times \text{0,78}}=\text{6,9}\%$$

One can see that in the case of a negative result of T_α_, the hypothesis H^+^ will be refuted (probability value of hypothesis is 6,9%, i.e., under test threshold), while the positive result of T_α_ will not increase the probability of the H^+^ hypothesis above 90%, which was the assumed value for the treatment threshold. However, the positive result of test T_*α*_ entails that hypothesis H^+^ has changed from an unlikely one that is tested by using simple tests to the one that deserves more attention and is worth confirming by using more advanced and expensive technics, even at the risk of the patient’s discomfort or various iatrogenic complications. Suppose that for a given patient, the result of T_*α*_ is positive.

The physician can decide to re-use the Bayesian Theorem when planning the next steps of their diagnostic strategy. Assume that the physician considers using test T_*β*_ after the positive result of T_*α*_. The frequency of disease D in the population has not changed. There are two ways of estimating the pre-test probability for a patient in case of T_*β*_ test. The first one is to assume the same value of the pre-test probability as for T_*α*_ test (15%). This seems justified because the frequency of disease D in the population, which includes the patient has not changed.

Suppose also that on the basis of the statistical data, the physician knows that the diagnostic sensitivity of test T_*β*_ is 84% and its diagnostic specificity is 96%. Then, using Bayes’ Theorem it is easy to find that the positive result of test T_*β*_, with the assumed pre-test probability, will increase the diagnostic probability of H^+^ from 15 to 78%. See below.

$$P\left({H}^{+}/{T}_{\beta }^{+}\right)=\frac{\text{0,15}\times \text{0,84}}{\left(\text{0,15}\times \text{0,84}\right)+\text{0,85}\times (1-\text{0,96})}=78\%$$

As can be seen in the above example, the assumed treatment threshold (90%) will not be exceeded and it *will not be possible* to implement the therapy.

However, notice that the diagnostician’s knowledge of the patient’s condition, after the positive result of T_*α*_ test has changed. This is a case in which “[…] one’s old posterior probabilities become one’s new prior probabilities” [39, p. 361]. The process of updating new information is called conditionalization. In light of this result, the doctor’s conviction that the patient actually has disease D has increased to 35%. Assuming the previous values of diagnostic sensitivity and diagnostic specificity respectively as of 84% and 96%, it can be calculated that the positive result of test T_*β*_, after the positive result from test T_*α*_, will increase the diagnostic probability of H^+^ from 35 to 92%. See below.

$$P\left({H}^{+}/{T}_{\beta }^{+}\right)=\frac{\text{0,35}\times \text{0,84}}{\left(\text{0,35}\times \text{0,84}\right)+\text{0,65}\times (1-\text{0,96})}=92\%$$

As can be seen in the above example, the assumed treatment threshold (90%) will be exceeded and it will now be *advisable* to implement the therap﻿y.

The pre-test probability value of 35% corresponds to the diagnostician’s knowledge about the T_α_ result. During conditionalization, one updates one’s degrees of belief after learning something new [39, p. 363]. This is the subjective dimension of the pre-test probability. However, in this case the subjectivity is of moderate nature. It is reasonable to assume that the value of the pre-test probability will be respected by doctors in further steps of the diagnostic process. This means that the condition of intersubjective probability is accepted.

## Simplifying the Bayesian method for the needs of medical practice

The analysis made in the previous paragraph may raise some concerns. What is the usefulness of such findings in real medical practice? Perhaps one would be inclined to agree that the physician knows the values of diagnostic sensitivity and specificity from the medical literature. However, it is difficult to imagine a physician who would carry out calculations designing a multi-stage Bayesian strategy. This may be the case in large clinical centers specializing in the differential diagnosis of certain types of diseases. However, the application of the Bayesian strategy seems to be beyond the reach of general practitioners and specialists admitting patients in smaller centers, where the greater diversity of patients’ complaints makes it impossible to develop a uniform diagnostic procedure. These reservations, however, are not accurate. Even general practitioners have a relatively simple method of determining the final probability value in Bayesian analysis without the formal calculus.

When looking for information characterizing diagnostic tests, doctors use publications in electronic databases. In the medical articles, the main parameters of the diagnostic tests – i.e., the diagnostic sensitivity and the diagnostic specificity – are usually combined and presented as a separate parameter called as the likelihood ratio (LR). This parameter is specified as follows.

$${LR}^{+}=\frac{diag.sensitivity}{1-diag.specificity}$$

One can see that LR determines the relationship between the diagnostic sensitivity and specificity. The diagnosticians know the LR value for a given test from medical literature and they also know the pre-test probability of the disease occurrence in the population (e.g., the prevalence rate). Thus, they can determine the post-test probability of a hypothesis using the so-called Fagan’s nomogram [40–42].

The Fagan’s nomogram is a type of the graph with three columns: the pre-test probability value, the LR value, and the post-test probability value. By connecting with a straight line the value of pre-test probability with the value of LR, one can determine the value of the post-test probability and then used it as the pre-test probability value for the next test.

Now, I will apply the above findings to the example analyzed in the previous paragraph. It is easy to ascertain that in the discussed example, for the positive results of the T_α_ test and T_β_ test, the values of LR will be respectively:

$$LR\left({T}_{\alpha }\right)= \frac{diag. sens}{1-diag. speci.}=\frac{\text{0,67}}{1-\text{0,78}}=\text{3,05}$$

$$LR\left({T}_{\beta }\right)= \frac{diag. sens}{1-diag. speci.}=\frac{\text{0,84}}{1-\text{0,96}}=21$$

Thus, the graph on the Fagan’s nomogram has the following form: Scheme 3

**Figure:**
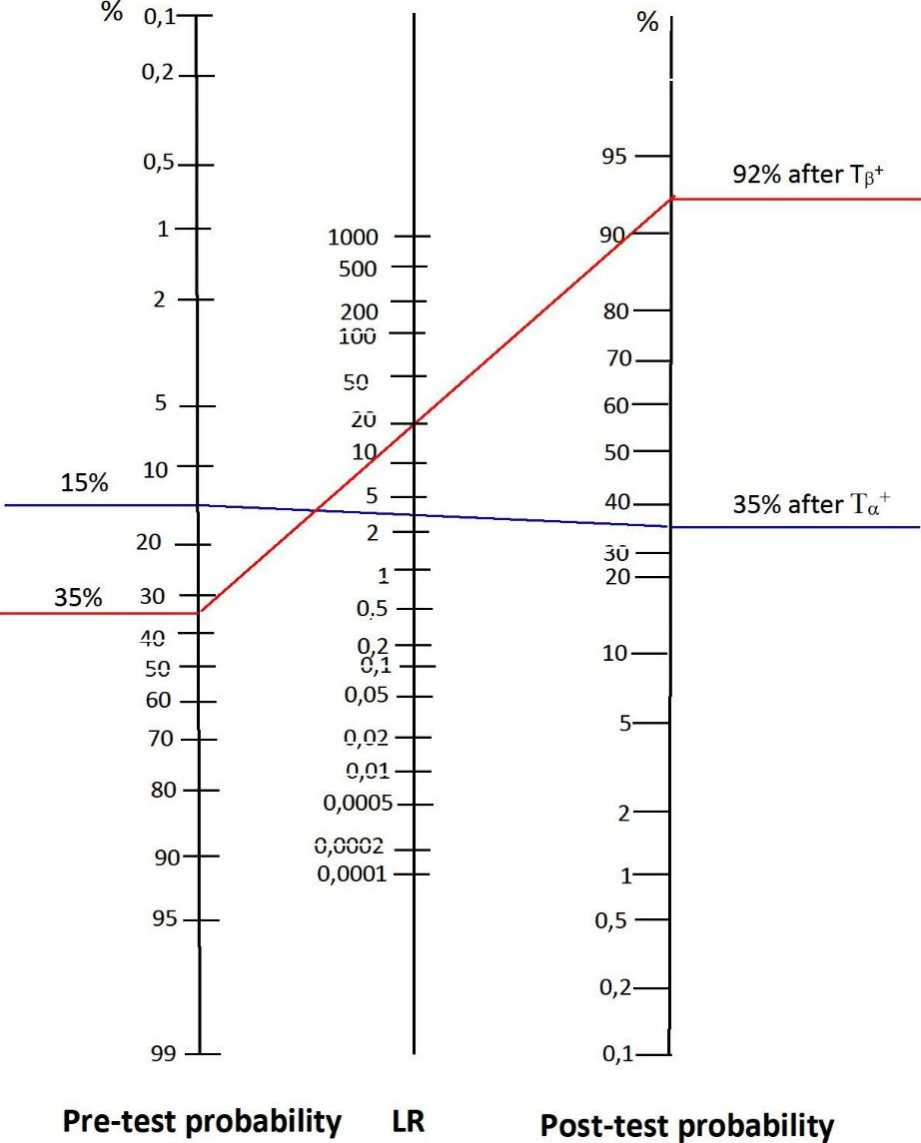
Scheme 3

The blue line connects the prior probability value of the H^+^ (15%) with the LR^+^ value (3,05) for the test T_α_^+^. The post-test probability after T_α_^+^ is 35%. The result obtained is then taken as the pre-test probability value for test T_β_^+^, for which LR^+^ = 21 (red line). The post-test probability after T_β_^+^ is 92%. 15

By using the Fagan’s nomogram, the diagnostician can therefore easily determine how the probability of a hypothesis will change in the subsequent stages of medical diagnostics. Hence, it is a tool that allows one to establish quick paths of diagnostic proceedings in the process of confirming or rejecting a hypothesis.

## Bayesian probability tree and pre-test probability problem reevaluated

Using Fagan’s nomogram allows for the construction of a probabilistic tree as a basis for designing diagnostic strategies. In the case of the aforementioned example, the Bayesian probabilistic tree has the following form. Scheme 4

**Figure:**
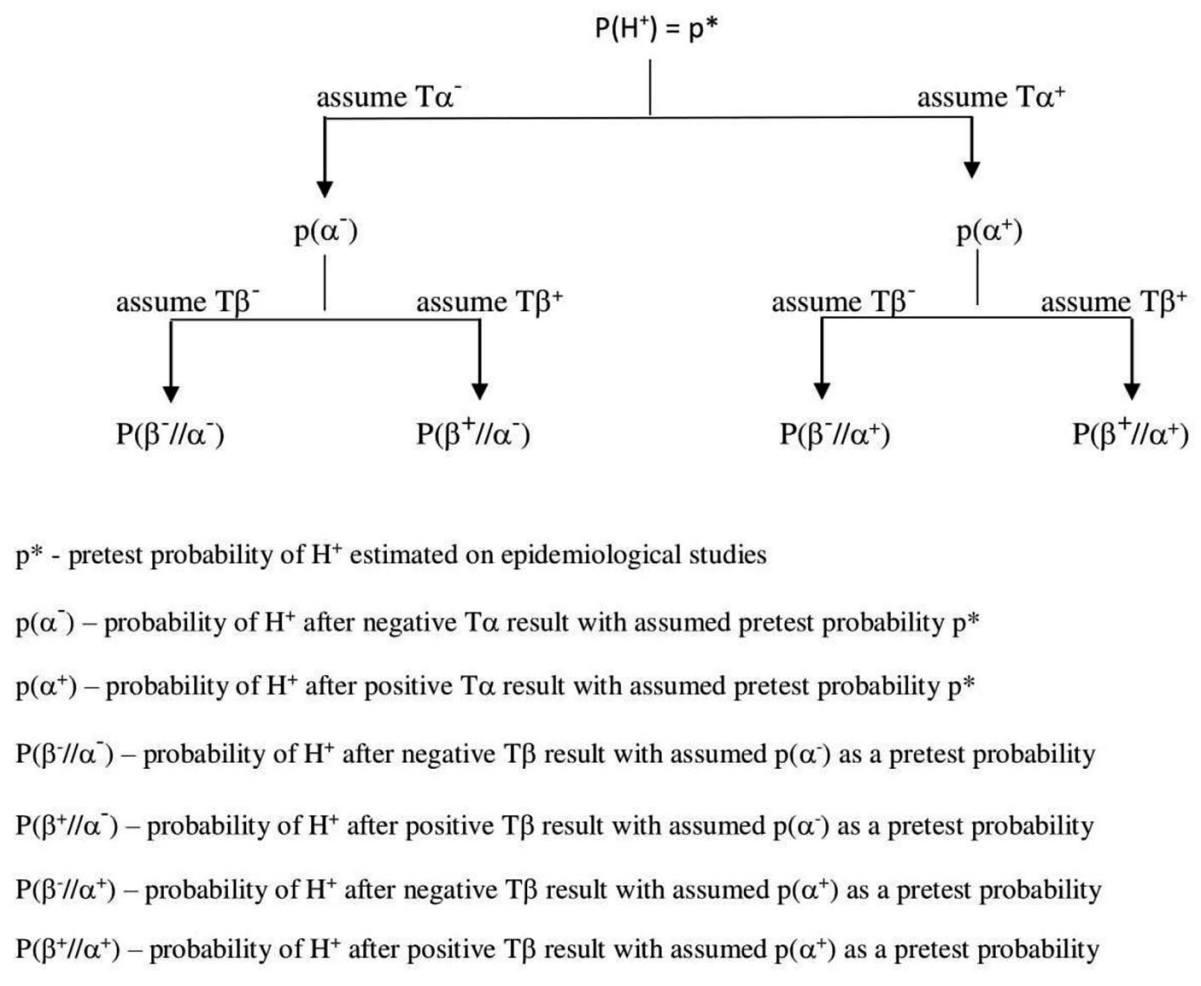
**Scheme 4**

In its general form, the Bayesian probabilistic tree shows how the post-test probability of the diagnostic hypothesis will change in the next stages of diagnostic process. Now consider whether anywhere in the tree it is necessary to accept a radically personalistic interpretation of probability.

There is no doubt that as far as possible (i.e., if there is sufficient epidemiological data), established on the basis of reference class, the pre-test probability value of the H hypothesis for the T_*α*_ test is specified as the frequency probability (e.g., as a prevalence value).

In the subsequent stages of the diagnostic process, the physician may use one of the two aforementioned methods for determining the pre-test probability value. On the one hand, they can take the post-test probability value from the previous test, as pre-test probability value for the next tests, according to scheme 4. The other method would be to abandon the Bayesian analysis presented in scheme 4.

Looking closer at this issue, assume that the physician is considering T_*α*_ test for patient A, in whom the disease is suspected. Note that if the positive result of the T_*α*_ test is to credibly increase the probability of the disease in a given patient, the following condition of test rationality (TR) must be met:

$${\text{TR:}}\,{\text{P(T + /H + )}}\,{\text{ > }}\,{\text{P(T + /H - )}}$$

This means that for a given reference class the probability of the positive result for the disease patients is greater than the probability of the positive results for healthy patients. This condition determines the rationality of performing a given diagnostic test (in order to confirm the disease hypothesis). If this condition is met, then the physician knows that the positive test result increases the probability of the disease on the basis of the frequency probability. On the other hand, if one recognizes that the test does not meet this condition, then, of course, there is no reason to conduct this test. The positive result, which would be more characteristic for healthy people is unreliable for the purpose of the disease identification.

When researchers postulate that one abandon the Bayesian analysis, it is recognized that the result of the earlier test (e.g. T_*α*_ ) does not compel the physicians to change their knowledge about the probability of the disease. In this situation, the value of pre-test probability for the subsequent tests should be assigned in consistency with the basic epidemiological data. Consequently, one can expect that physicians should accept the condition of the test’s rationality, and at the same time one can recognize that the test result may not affect their beliefs. The demand to discard the Bayesian analysis leads to a situation in which an agent is not willing to accept new data, which violates the intersubjective probability condition.

## The need for intersubjective probability in medical diagnosis

The decision-making process in medical diagnosis has been the subject of many analyses. For a long time, it has been maintained that the credibility of decisions made in this process is guaranteed by the frequency interpretation of probability. With the development of the EBM approach, attention has been drawn to the use Bayes’ Theorem in the process of designing diagnostic strategies.

The possibility of using Bayes’ Theorem in the field of medical diagnostics raises the problem of the credibility of this cognitive method. Various criticisms formulated against the Bayesian analysis are well known. It is commonly maintained that using the Theorem involves accepting the personalistic interpretation of probability. The thesis of the present article has it that this conviction is harmful from the perspective of the patient, because it inhibits the use of valuable cognitive tools in the diagnostic process.

The present paper has aimed to show that calculating the post-test probabilities in the consecutive stages of the medical diagnostic process with the use of Bayes’ Theorem does not require accepting the strongly personalistic interpretation of probability. This paper has argued that two dimensions of subjectivity have to be distinguished in the philosophical controversy over the Bayesian analysis. The first one concerns the radical personalistic view in which the probability value is established arbitrary by the agent independently of the frequencies of the events. When making a decision in a risk situation, the agent makes a bet in which they accept rates on the basis of their analysis of gains and losses. The second dimension of subjectivity in the probability interpretation is of more moderate nature. In this approach, it is assumed that agents may assign different probability values to a given event, if they do not have the same data. Otherwise, they would assign the same probability. The approach is based on the concept of intersubjective probability proposed by D. Gilles. In the present article, it has been argued that this interpretation is particularly appropriate for estimating the reliability of Bayesian analyses in the field of medical diagnosis. What is the reason for defending the intersubjective interpretation in the discussion of Bayesian analysis?

First, it seems reasonable to think that, for practical reasons, diagnosticians seek intersubjective data during the diagnostic process. Bayesian analysis based on the intersubjective probability interpretation offers a simple and effective method for making diagnostic medical decisions. As Sprenger pointed out, many statisticians use Bayesian methods as a technical tool, however they would not qualify themselves as subjectivists [43, p. 402]. At the first stage, the clinicians look for epidemiological studies on the pre-test probability, or they recall any previous cases of the frequency probability which are well known by experts. At the consecutive stages of the diagnosis process, they use various post-test probabilities calculated previously as pre-test probabilities for later tests. Thus, diagnosticians obtain a tool for designing multi-stage diagnostic strategies by what cannot be achieved with other popular methods (e.g., by using the predictive parameter).

Notice that the intersubjective interpretation of probability also pays attention to some important epistemological issues. The first one is the reliability of the information flow. Generally, the reliability of information flow is the idea that disseminating information about the pre-test probabilities is effective. This means that every potentially interested agent can access the information. That is, they know how to find it and how to apply the appropriate tools (e.g., data bases and computer programs) which allows one to design multi-stage Bayesian probability trees.

The Bayesian approach, when based on the intersubjective interpretation, guarantees a cumulative growth of knowledge about the patient during the subsequent stages of the diagnostic process. It is particularly important in case of the diagnostic process, which takes place or is improved in different branches of medical specialties. In these cases, when a patient is treated by different doctors, confidence in the previous tests’ results is needed. Specialists in the subsequent stages of medical diagnosis must believe that there have been reliable reasons for the previous diagnostic procedures. The open question is whether the *justification* for these activities should be known for other diagnosticians. It seems that, due to the high degree of medical specialization, it would not be reasonable to expect that diagnosticians always know the justification(s) for the activities undertaken by their predecessors. At the minimum, they should be assured that scientific justification for these activities would be given if necessary. The agent expects that the information about the pre-test probability value has been subjected to a severe control by qualified experts.

## Conclusion

This paper has argued that the perspective of epistemological analysis of the Bayesian method must be changed from a radical personalistic account to a moderate account. There is a strong need to discuss the pre-test probability problem, for which the intersubjective interpretation, according to the moderate personalistic account of the Bayesian method, could be the point of departure. Ultimately, it needs to ascertained how one can improve the ways of estimating the intersubjective probability in the field of medical practice.

## Footnotes

1 A separate issue is the real practice of informal inferences carried out by diagnosticians. However, this problem is not a subject of analyses conducted in the article.

2 Keynes refers to it as the “Principle of Indifference.”

3 The interpretation based on the NSR principle was labeled by Savage as “necessarian” or “gambler’s” probability, which is of symmetrical nature, because it assigns the same probability value to all possibilities.

4 There are at least several paradoxes of the NSR principle that are discussed in the literature. Generally, these paradoxes show that when using the NSR principle, one can assign probability value in such a mode that the sum of the mutually exclusive possibilities will be higher than 1 [15, p. 37–42].

5 The problem of single event probability estimations is extensively discussed in contemporary philosophical analysis [18].

6 See also [22, p. 68].

7 The example is a development of the famous case given by Ramsey: “Whenever we go to the station we are in a sense betting that a train will really run, and if we had not sufficient degree of belief in this we should decline the bet and stay at home” [27, p. 183].

8 The obvious question is whether the diagnostician correctly understands the diagnostic concepts; see [34].

9 Determining the diagnostic specificity and the sensitivity causes several problems. For one, their values are not constant and they depend on the disease development progress[35, 36].

10 There is no space here to discuss the epistemological problems involved in determining these parameters.

11 The concept of “really ill/healthy” should be understood as “ill/healthy according to the standard established by reference test results”.

12 Diagnostic sensitivity and diagnostic specificity must be distinguished from analytical sensitivity and analytical specificity. The latter two refer to the laboratory method. In the next part of this article, the concepts of sensitivity and specificity will be used only to characterize the diagnostic test, not the laboratory method.

13 In clinical practice, this is not obvious because of the so-called subadditivity problem. The subadditivity of the pre-test probability means that the sum of probabilities considered by the diagnostician for a single case scenario exceeds 100% [37].

14 Another problem with the pre-test probability value estimation is that the value is very often approximately characterized as “ranges” or “intervals.” This problem, however, is rather technical and differs from the philosophical issue of the subjective probability evaluation [9].

15 The above diagram is intended only to illustrate the method but cannot be used to make precise determinations of the hypothesis probability value in the real diagnostic process.
